# Correlation of Cutaneous Manifestations With Body Mass Index (BMI) in Polycystic Ovary Syndrome (PCOS) Patients in a Tertiary Care Centre: An Observational Study

**DOI:** 10.7759/cureus.20695

**Published:** 2021-12-25

**Authors:** Pramila Jena, Malvika Tiwari, Soumya R Panda, Subhra Samantroy, Jyochnamayi Panda

**Affiliations:** 1 Obstetrics and Gynecology, Kalinga Institute of Medical Sciences, Bhubaneswar, IND; 2 Obstetrics and Gynecology, Ramakrishna Mission Seva Pratishthan, Kolkata, IND

**Keywords:** alopecia, hirsutism, pcos, acne, bmi

## Abstract

Background: Polycystic ovary syndrome (PCOS) is a common disorder affecting mostly reproductive age group women. It is characterized by clinical and biochemical evidence of anovulation, hyperandrogenism and a polycystic ovary in the ultrasound. The aim of the present study is to find out the prevalence of cutaneous manifestations in PCOS and the correlation of their cutaneous manifestations with body mass index (BMI).

Materials and Methods: A hospital-based observational, prospective study was conducted with 251 patients over a period of 18 months. Patients were divided into two groups based on their BMI values (more than 25 and less than 25) and screened for cutaneous manifestations. Correlation between the cutaneous manifestations and BMI was noted.

Results: Maximum number of manifestations were seen in overweight patients (n=189). Hirsutism was the most common cutaneous manifestation followed by acne. But the only statistically significant association noted in the present study was between acne and BMI (p-value 0.009).

Conclusion: PCOS is a common disorder seen in females of the reproductive age group. Though the prevalence of cutaneous manifestations was more commonly seen in overweight (BMI between 25 and 30) and obese women (BMI >30), they were also found in lean groups. Hence evaluation and counselling regarding lifestyle modification are important not only for obese but also in lean PCOS.

## Introduction

Polycystic ovary syndrome (PCOS) is a multisystem disorder affecting around 4%-20% of reproductive age group women and involves reproductive, endocrine and metabolic systems [1]. It is characterised by anovulation, clinical (acne, virilisation, etc.) and biochemical evidence (increased levels of serum testosterone, androstenedione, etc.) of hyperandrogenism with or without a polycystic ovary in the ultrasound. These patients are found to be at increased risk of infertility, dysfunctional bleeding, endometrial carcinoma, obesity, type II diabetes mellitus, dyslipidemia, hypertension and, possibly, cardiovascular diseases [2].

The pathophysiology of PCOS is multifactorial involving defects in steroid synthesis and metabolism leading to increased production of ovarian androgens. Defects in weight and energy regulation may result in obesity. There have been many studies evaluating the relationship between obesity and PCOS [3-5], cutaneous manifestations and PCOS [6-8], but there is a paucity of literature relating cutaneous manifestation with body mass index (BMI). Our study aimed to correlate the cutaneous manifestations of PCOS women in various BMI groups. These cutaneous manifestations may constitute a visible marker for a possible underlying metabolic and endocrine problem and such cases may be targeted for long-term follow-up for early detection and management of such disorders.

## Materials and methods

Study design: This was a hospital-based observational, prospective study conducted over a period of 18 months in the Department of Obstetrics and Gynecology at Pradyumna Bal Memorial Hospital, Kalinga Institute of Medical Science, Bhubaneswar, Orissa. Institutional ethics committee approval was obtained with approval number - KIMS/KIIT/IEC/160/2018.

Objective: The objective of this study was to correlate the cutaneous manifestations with BMI in women with PCOS.

Inclusion criteria: Patients of reproductive age group (between 20 and 40 years of age) diagnosed with PCOS as per the Rotterdam criteria were included in the study after obtaining an informed written consent.

Exclusion criteria: Pregnant women, those with the presence of any other endocrinopathy (hyperprolactinemia, uncontrolled thyroid disorder, Cushing syndrome, hypothalamic, pituitary, or adrenal disease, etc.), participants with age less than 20 years or more than 40 years, those on hormonal medications or drugs for any other chronic illness (except for hypothyroidism) during the two months preceding the study and those who did not give consent were excluded from this study.

Data collection: A total of 251 diagnosed cases of PCOS, of the reproductive age group (20-40 years), attending our OPD were included in this study. Once patients were enrolled for the study, a detailed medical history was taken in each case, with special emphasis on various cutaneous complaints including their duration, evolution and progression. General physical examination, systemic examination, breast and pelvic examination, as well as a detailed dermatological examination, were carried out. They were screened for signs of hyperandrogenism such as acne vulgaris, hirsutism, seborrhoea, acanthosis nigricans, hair loss due to androgenic alopecia and striae distensae. For cutaneous manifestations, their respective scoring system and grading systems were applied. Investigations (thyroid function tests, and abdominal ultrasound) were done. Height (in metres) was measured without shoes against a wall-fixed tape and weight (in kilograms) with light clothing and without shoes on a platform scale with a 1.5kg subtraction to correct for clothing weight. Waist circumference was measured in centimetres at the level of the umbilicus, without clothing and in standing position. Hip circumference was measured in centimetres at the level of greater trochanter. Waist-to-hip (W/H) ratios were calculated for each participant. These women were further divided into two groups according to their BMI - (kg/m^2^) and weight (height)^2^. Those with a BMI of >25 (obese and overweight) were grouped as Group A and those with a BMI of <24.9 (non-obese) were grouped as Group B.

Statistical analysis

The data were tabulated and expressed as mean ± SD for continuous variables or frequency and percentage for categorical variables. Chi-square or Fisher Exact test was used to determine the association between two categorical variables. The student’s t-test was performed to test the significance of the difference between the two groups. All statistical calculations were performed using the SPSS software version 21 (SPSS Inc., Chicago, IL, USA), and a p-value ≤0.05 was considered statistically significant.

## Results

The majority of the patients in our study were in the age group of 20-29 years (98.01%), with a mean age of 25.7 years for the study subjects. The study population was divided into two groups based on BMI. Group A (n=167) constituted participants with BMI >25 and group B (n=84) constituted those with BMI of less than or equal to 24.9. The entire study population constituted 3.5% obese, 62.94% of overweight, 32.66% of normal BMI and 0.7% of the underweight population (Table 1).

**Table 1:** Distribution of patients based on body mass index and age group

	Frequency	Percentage (%)
Age groups		
10-19	33	21.85
20-29	148	98.01
30-39	66	43.71
40-50	4	2.65
Body mass index		
Underweight	2	0.7
Normal body weight	82	32.66
Overweight	158	62.94
Obese	9	3.5

Around 75.3% (189 out of 251) of participants had cutaneous manifestations in form of acne, hirsutism, alopecia and acanthosis nigricans. About 41.80% had single cutaneous complaints while 54.5% of patients presented to us with a minimum of two cutaneous manifestations. The detailed incidence of cutaneous manifestations is shown in Table 2.

**Table 2:** Association of BMI with the type of cutaneous manifestations BMI: Body Mass Index

BMI groups						
	Underweight (N[%])	Normal body weight (N[%])	Overweight (N[%])	Obese (N[%])	Total	P-value
Cutaneous Manifestations						
Acne	0	47 (37.3)	71 (56.35)	8 (6.35)	126 (100)	0.009
Hirsutism	0	32 (30.19)	72 (67.92)	2 (1.89)	106 (100)	0.303
Alopecia	1 (2.63)	16 (42.11)	20 (52.63)	1 (2.63)	38 (100)	0.742
Acanthosis nigricans	0	5 (29.41)	11 (64.71)	1 (5.88)	17 (100)	0.512
Acne, hirsutism	0	14 (35)	24 (60)	2 (5)	40 (100)	-
Acne, alopecia	1 (2.7)	16 (42.1)	20 (52.6)	1 (2.70)	38 (100)	-
Acne, acanthosis	0	1 (25)	2 (50)	1 (25)	04 (100)	-
Hirsutism, acanthosis	0	1 (16.67)	5 (83.33)	0	06 (100)	-
Hirsutism alopecia	0	4 (40)	6 (60)	0	10 (100)	-
Alopecia, acanthosis	0	2 (33.33)	4 (66.67)	0	06 (100)	-
Acne, hirsutism, acanthosis	0	2 (66.67)	1 (33.33)	0	03 (100)	-
Acne, hirsutism, alopecia	0	2 (50)	2 (50)	0	04 (100)	-
Total	02	82	158	09		-

The most common association of cutaneous manifestations was acne and hirsutism in 15.9% followed by acne and alopecia in 14.7% of patients. About 3.7% of patients had more than two cutaneous manifestations. Out of a total of 189 women with cutaneous manifestations, acne was the most common complaint (50.1%) followed by hirsutism (42.2%), alopecia (15%) and acanthosis nigricans (6.7%).

Amongst our study population, maximum cutaneous manifestations were seen in overweight followed by obese category. Overweight patients had hirsutism (67.92%) as the most common cutaneous manifestation followed by acne. In obese populations, acne (6.35%) was the most common cutaneous manifestation. Even in the normal BMI population, acne (37.3%) was the most common followed by hirsutism and alopecia. Amongst underweight patients, alopecia was found to be the sole cutaneous manifestation in this study (Table 2).

In the present study, although the association between acne and BMI was found to be statistically significant (P-value - 0.009), there was no significant correlation between the cutaneous manifestations and BMI (Table 3).

**Table 3:** Correlation of cutaneous manifestations with BMI * Correlation is significant at the 0.01 level (2-tailed). BMI: Body Mass Index

Cutaneous manifestations	Correlation coefficient	P-value*
Acne	-0.065	0.304
Hirsutism	0.062	0.325
Acanthosis nigricans	0.019	0.768

## Discussion

Our study included 251 women diagnosed with PCOS according to Rotterdam’s criteria, which include two of the three findings - polycystic ovaries, anovulatory cycles and hyperandrogenism. [9]. Amongst them, most of the patients were in the age group of 20-29 years (98.01%) with a mean age of 25.7 years. Overweight and obese cases constituted around 62.94% and 3.5% of the total study subjects, respectively. In contrast, several other studies found a majority of PCOS women as obese [3,4,10-12]. The overall incidence of cutaneous manifestations (75.3%) in our study was found similar to that of Majumdar and Singh [3], and Sachdeva et al. [5]. Amongst all cutaneous manifestations, we found acne to be the most common one (50.1%) followed by hirsutism (42.2%), alopecia (15%) and acanthosis nigricans (6.7%) in our study. This is in line with the results found by Sharma et al. (64%) who also found acne as the most common cutaneous manifestation [13]. Jain et al. found hirsutism as the most common dermatologic manifestaion [6]. However similar to many other studies, we observed hirsutism in 42.2% of patients [7,8].

Various studies have proved clinical hyperandrogenism was significantly higher in obese as compared to non-obese PCOS women [3,5,7]. In our study, we also noticed majority of cutaneous manifestations in overweight group than in the lean group (66.5% vs 33.4%), but these findings were not found statistically significant (p-value = 0.07). This higher incidence of cutaneous manifestations in the obese group could be due to the fact that obesity results in an increase in the androgens and a decrease in sex hormone-binding globulin (SHBG) levels, thus increasing free androgen level. When we compared the cutaneous manifestation in BMI subgroups (overweight versus lean), we found majority of the patients in the overweight sub-group had hirsutism (67.92% vs 30.19%) followed by acne (56.35% vs 37.3%). These results were in line with other studies as well [14,15]. In our study, although acne was found to be significantly associated with high BMI (p-value -0.009), we did not find any significant statistical correlation between BMI and cutaneous manifestations. This suggests that high BMI is an independent risk factor for development of future metabolic and endocrine problems irrespective of the presence of cutaneous manifestations. However, the poor correlation of BMI possibly indicates that apart from obesity, cutaneous manifestations of PCOS may have a complex link with some other external and internal factors such as disturbed hormonal milieu, habit of junk food intake, poor skin hygiene, etc. These factors have to be evaluated and clarified in future studies.

Limitations

The limitation of the present study includes a smaller sample size. Moreover, a long-term follow-up was not done which may throw some more light on the association of cutaneous marker with future development of metabolic and endocrine problems.

## Conclusions

PCOS is a reproductive-endocrine-metabolic disorder affecting the entire lifespan of a woman with the future risk of type 2 diabetes, cardiovascular problems. The dermatologic manifestations play a significant role in the diagnosis of PCOS and may serve as a cutaneous marker to endocrine-metabolic abnormalities. Although these are more prevalent in overweight and obese group, we did not find any correlation of cutaneous manifestations with BMI. This emphasizes that those with high BMI should be counselled regarding lifestyle modifications irrespective of the presence of cutaneous manifestations. This can be of great help in preventing or delaying the onset of metabolic and other long-term complications. We recommend future studies to evaluate the association of these cutaneous markers to long-term metabolic and endocrine problems.
